# Behavioral Biases and Judicial Decision-Making in Brazil

**DOI:** 10.3390/bs14100922

**Published:** 2024-10-10

**Authors:** Benjamin Miranda Tabak, Liziane Angelotti Meira, Ana Clarissa Masuko dos Santos Araujo, Aline Guiotti Garcia

**Affiliations:** Getulio Vargas Foundation, School of Public Policy and Government (FGV/EPPG), Brasilia 70830-020, Brazil; liziane.meira@fgv.br (L.A.M.); ana.masuko@fgv.br (A.C.M.d.S.A.); aline@dradv.com.br (A.G.G.)

**Keywords:** decision-making, behavioral insights, impartiality, judicial biases, cognitive biases, behavioral economics, experimental economics, nudges, narrative review

## Abstract

We identify and present Brazil’s most common behavioral and heuristic biases in judicial decision-making. Through bibliographic and specific cases, we notice the occurrence of the representativeness heuristic, availability heuristic, anchoring heuristic (anchoring effect), confirmation bias, and affect heuristic bias in Brazilian judicial decisions. We also present the current state of Brazilian legislation and its amendments that aim at impartiality in the production, the assessment of evidence, and the judge’s conviction. Finally, we present the suggestions and initiatives that aim to mitigate biases and heuristics in judicial decision-making in Brazil, especially with awareness techniques, the replacement of judges by algorithms, and the review of judicial decisions by collegiate bodies.

## 1. Introduction

The capacity for human rationality has limits. The Theory of Bounded Rationality, through which the behavior of the human being, instead of maximizing their decision (after analyzing all the variables, making the best decision possible), suggests that people have bounds and do not maximize and always make optimal decisions. On the contrary, most of the time they make sub-optimal decisions [1]. To what extent are judges expected to demonstrate complete impartiality as mandated by the law? Are they not subject to the cognitive failures that affect human beings?

The literature on judgment and decision-making has grown in Brazil [2,3]. After all, are the judges utterly exempt from any bias during the assessment of the evidence to render the decision? Do social and political prejudices tend to influence the decision?

Given these inquiries, the growing body of literature on the topic has revealed that, irrespective of legislative prescriptions, judges, being human beings, are susceptible to cognitive biases (See [4,5,6,7]. However, to what extent do these biases undermine the desired justice and impartiality sought by the parties? When the State is part of the process, is there a greater tendency to decide in its favor? In Brazilian Labor Law, is there a greater tendency for the judge to favor the employee?

The Brazilian legal system prescribes rules for assessing evidence so that they are not based solely on the judge’s perception. There is a review of judicial decisions by collegiate bodies but only when one of the parties appeals. However, in addition to legal requirements, there are initiatives to raise awareness among judges.

This work analyzes the influence of behavioral biases on decision-making by judges in the Brazilian judicial context. Its general objective is to identify the most common biases in judicial decisions from the existing literature in Brazil, assess their presence in judicial decisions, and assess the possibility of the absence of an evaluation of the evidence due to the heuristics and biases verified. Finally, we present suggestions and initiatives to combat or reduce behavioral biases in judicial decision-making.

Based on the Theory of Decision Making, Behavioral Sciences, Behavioral Economics, and the General Theory of Law and also considering the evolution of the debate about judicial decision-making and behavioral biases through the bibliographic documents, the hypothesis initially analyzed is that behavioral biases (especially the heuristics of availability, representativeness, anchoring, and affection as well as confirmation bias) greatly influence judicial decision-making, according to the existing literature. However, even though such influence has been demonstrated and there are some initiatives, none effectively managed to reduce the biases in Brazilian judicial decisions.

Therefore, given that the influence of behavioral biases by the existing literature is considered inevitable and inescapable, the present work contributes to the literature by highlighting the most common heuristics and behavioral biases in judicial decision-making and strategies to combat or reduce them.

In Brazil, decisions are always made by single judges who must motivate their decisions in writing (required by law). Defendants can defend themselves by appealing individual decisions made by judges to courts that rule as a collegiate body. So, our analysis focuses on judges’ individual decisions, which can suffer from cognitive biases. We will then discuss how collegiate bodies can mitigate these biases in individual decisions.

Most of the literature on behavioral economics focuses on laypeople and how cognitive biases may worsen their individual decisions. This may happen because they lack the expertise necessary to make these specific decisions, such as a lack of knowledge of probability. Our focus is on legal decisions in which the decision-makers are experts in using the law. Therefore, we should not expect cognitive biases to be an essential issue regarding decision-making. However, recent research has reviewed professional decision-making in different areas and finds that cognitive biases impair decision-making [8].

This article is divided into four sections addressing the main points related to biases and heuristics in Brazilian judicial decisions, their influence on the production of evidence, and suggestions for mitigation or elimination. Section 1 presents the most common biases and heuristics in Brazilian judicial decisions, emphasizing the representativeness heuristic, availability heuristic, anchoring heuristic (anchoring effect), confirmation bias, affect heuristic, coherence, and result bias. Section 2 discusses evidence production, assessment, and the judge’s conviction in the Brazilian legal system. Section 3 presents the suggestions and initiatives that aim to combat or reduce biases and heuristics in judicial decision-making in Brazil, especially using awareness techniques, the replacement of judges by algorithms, and the review of judicial decisions by collegiate bodies. Finally, based on the literature studied, the most common biases and heuristics in Brazilian judicial decisions and the existing initiatives to combat them are identified. The last section presents the financial considerations.

## 2. Materials and Methods

Refs. [9,10] identified that human rationality has limits. Therefore, they created the Theory of Bounded Rationality. However, the literature has advanced and demonstrated that the rational human capacity makes mistakes in addition to limiting rationality. In general, when it comes to decision-making, people often rely on intuitions (biases) to facilitate and speed up thinking, using specific shortcuts (heuristics) to decide. As [11,12] mentioned heuristics may be quite useful, but sometimes they can lead to severe and systematic errors.

In this way, overcoming the Theory of Rationality was once again proven since human beings do not analyze all probabilities in decision-making. According to the heuristics, even if the human being wished and could, they would not impartially evaluate all variables and possibilities in decision-making. Behavioral economics is intrinsic to human beings.

As [12] warned, trust in cognitive biases arising from judgment heuristics is not restricted to laypeople but to any human being. The most experienced researcher, although aware of the influence of biases and heuristics when thinking intuitively, will also be prone to them. Therefore, should we assume that judges are impartial in decision-making in a process or that they value the evidence impartially?

Ref. [12] states that tired judges make the easiest decision. Generally, people are risk-averse and analyze, to some extent, the consequences of their choices. However, there is a risk for the judge when making a judicial decision, as the parties will experience the results, and depending on the results, judges may suffer from violence or even death threats. Loss-averse or risk-averse judges may suffer more from this influence [11,12]. Nonetheless, it is quite difficult to ascertain how these potential threats may influence judicial decision-making, and they are more likely in very specific situations in which the stakes may be very high. Therefore, we will focus on specific cognitive biases that may impact judicial decision-making on a regular basis.

On the other hand, the literature has identified other behavioral biases in judicial decisions, which could interfere with the impartiality prescribed by legislation. Verifying availability, representativeness, anchoring, affection, or confirmation bias is common [13].

In summary, heuristics correspond to rules that produce problem-solving and decision-making satisfactorily and fast for the individual. They are the shortcuts used by the brain that, instead of analyzing all the available information to choose the best path, take the best route, even if this can lead to an erroneous decision.

Ref. [11] present three types of heuristics: representativeness, availability, and anchoring/fitting. These heuristics can be found in judicial decision-making.

### 2.1. Representativeness Heuristic

The representativeness heuristic demonstrates the bias of human beings to make judgments based on probabilities, even if unrealistic. These errors in probability analysis based on the belonging of an object to a group, whether representative, similar, or stereotyped, can lead to erroneous decisions. Ref. [11], when approaching this heuristic, emphasize that it is the judgment of probability that can lead to severe errors because similarity, or even representativeness, may not be influenced by several factors that should affect probability judgments.

To exemplify the ease with which this heuristic is presented, Ref. [12] tells the story of Tom W and, later, of Linda and shows how, through a few characteristics, each character is quickly placed in a certain stereotype. From this conclusion, we deduce several other features (although that previous conclusion is questionable, and the individual knows this).

The author quotes two “sins” of the heuristics. The first one is the excessive predisposition to predict improbable events. For instance, by seeing a person sitting on a bench reading a newspaper, the observer soon imagines that the individual is an intelligent person with a higher chance of having a doctor’s degree than not possessing an undergraduate degree. The observer concludes this even knowing that people who do not have an undergraduate degree in Brazil are more abundant than those with a doctor’s degree. The observer completely ignores the actual probability (base rate), even if there is science in it.

However, the mere fact that someone reads the newspaper does not indicate their education, so the conclusion may be wrong. The second sin of representativeness is based on ignorance of the quality of evidence that gives rise to probability. After all, as the author states, the individual only knows “what you see is all there is” (WYSIATI). Therefore, the evidence they use is fragile and could lead them to wrong conclusions.

In this context, this heuristic can be manifested in judicial decisions, as can be observed in the excerpt from the habeas corpus judgment by the Court of Justice of São Paulo (Excerpt from Habeas Corpus no. 2301453-54.2022.8.26.0000, of January/2023):

“He emphasizes that the crime imputed to her occurred more than two decades ago and that there has been no repetition of the crime, demonstrating that the patient is an honest woman, a mother of a family, a good person, and presumably innocent”

(transcripted HC 2301453-54.2022.8.26.0000).

It emphasizes that the crime imputed to her occurred more than two decades ago, and there was no criminal reiteration, demonstrating that the defendant is an honest woman, a mother of a family, a good person, and presumed innocent. The defendant’s description as “an honest woman, mother of a family” leads to the conclusion of her innocence.

Likewise, as evidenced by [14], the representativeness heuristic is ingrained in judgments involving indigenous people. This is because, according to the authors, the classification of someone as a “true Indian”, “acculturated Indian”, or “integrated Indian” demonstrates the influence of the stereotype, even though it is not possible to deduce any assessment of the guilt of the supposed author of the crime (As an example, Ref. [14] cited: Habeas Corpus 85198, Rapporteur: Min. EROS GRAU, First Panel, judged on 11/17/2005, DJ 12/09/2005; Habeas Corpus 30.113/MA, Rapporteur Minister GILSON DIPP, FIFTH CLASS, judged on 10/05/2004, DJ 11/16/2004, p. 305).

The following are needed to avoid representativeness: (i) in the face of little evidence, the base rates should guide the probabilities and not the mere intuition of the individual; (ii) the decision-maker must question the diagnostic of this evidence. Therefore, the representativeness heuristic is primarily based on the evidence or evidence used in a case, although the judge cannot decide only through the probability measured by the base rate.

Therefore, there is no doubt about the topic’s relevance, notably about the perception of the availability heuristic in everyday life. However, it is difficult to construct empirical evidence of the influence of this heuristic on judicial decision-making. This is why we chose to present some arguments explaining how cognitive biases may impair judicial decision-making and present some specific cases in which these biases may occur.

### 2.2. Availability Heuristic

The availability heuristic is based on the ease of the individual to access mental contents, whether in retrieving memories or constructing ideas, which facilitates associations and, consequently, influences the decision-making process. To simplify the concept, Refs. [11,12] mention that there are several situations in which people assess the frequency of a class or the probability of an event by how easy it is for instances or occurrences to be recalled.

This is a valuable clue for estimating frequency or probability, as occurrences of broad classes are generally better and more easily remembered than less frequent ones. Among the examples brought by [12], the individual’s experience with the Judiciary will permanently influence them much more than any similar incident the individual has been aware of (regardless of the source). Availability, however, can also be influenced by, for example, the affect heuristic, as it has been found that people recurrently consult their emotions to make judgments and decisions. Such choices directly express their feelings and their typical behavior, although they do not realize it.

To exemplify the manifestation of this heuristic in judicial decisions, see the excerpt below from the conclusion of the Court of Justice of the State of São Paulo (Excerpt from judgment in case No. 1500145-66.2020.8.26.0491 in January/2022, Court of Justice of São Paulo, Rapporteur Alves Braga):

Undeniably, the word of the victim in crimes of sexual violence assumes exceptional relevance and must deserve credibility and prevail over the word of the accused when supported by other evidence in the file and enjoys a good reputation. Note that, according to the court’s jurisprudence, the word of the accused, who “has a good reputation”, will be influential in the trial.

Regarding public policies, Ref. [12] cites the name given by jurist Timur Kuran to the system that allows biases to be absorbed by public policies, the so-called availability cascade. In short, the idea is a self-sustaining cascade of events that can be initiated through media news. According to the author, a relatively minor event within the chain could cause the public to panic and give rise to large-scale government action. Thus, despite distrusting irrational fears arising from availability cascades, he also defends that such worries, even if irrational, should not be ignored by decision-makers, and public policy must combine experts’ knowledge with the population’s emotions and intuitions.

Therefore, how can we say such heuristics do not significantly affect judicial decision-makers? Considering the State’s success in several tax actions and the tax evasion of companies and entrepreneurs, when the judge is faced with tax enforcement proceedings, is there a greater tendency to decide in favor of the public entity? Through the availability heuristic, it is possible to confirm it.

### 2.3. Anchoring Heuristic (Anchoring Effect)

Refs. [11,12] clarify that several situations lead people to make estimates starting with an initial and erroneous value. This value must be adjusted to arrive at the final answer. However, the judgment made by the individual in the decision-making process will consider that initial value. Ref. [12] exemplifies that the asking price will influence you if you think how much you should pay for a house.

Ref. [12] presents two existing systems in the human mind, 1 and 2. According to the author, System 1 operates automatically and quickly with little effort and without voluntary control. System 2, however, is dedicated to the most strenuous mental activities. These activities are associated with experience, choice, and concentration. Thus, not without reason, the author claims that System 2 is the lazy controller. Regarding anchoring, there is a way in which a deliberate process of adjustment occurs for System 2, but there is also a way in which it happens through priming, directed to System 1.

Priming, arising from the associative effect, translates the idea that a word or thought can evoke another. For example, when hearing the word “day”, it is common for the individual to associate it with bright or sunny. Therefore, System 1 tries to build the idea that the anchor (the initial number) is natural.

As [12,15] warns, the effect is so powerful that even random anchors can be as influential as informative anchors. Acting first in a negotiation has a powerful impact by allowing for the initial anchor to be set. As an example, Ref. [12] recognizes the risk of developing a cap on compensation granted by judges in common cases in the Judiciary. Despite bringing some security to both parties, the cap would act as an anchor, eliminating all indemnities whose judicial analysis had decided for higher amounts and raising the value of many others that would otherwise be much smaller.

Finally, the author presents a study in which experienced German judges first read the description of a woman arrested for shoplifting and, later, observed the result of two falsified dice (which would always give a three or nine). Subsequently, the judges were asked whether they would sentence the woman to a term of imprisonment greater or less, in months, than the number given. According to the study, those who scored a nine on the dice said they would sentence her to 8 months; those who scored three would sentence her to 5 months. The reported anchoring effect was 50%.

For example, see the excerpt below (Excerpt from judgment in case No. 1503586-39.2018.8.26.0228 in June/2019, Court of Justice of São Paulo, Rapporteur Hoeppner Dutra):

“In crimes against customs, as a rule, the evidence is not a collection of facts, it is almost always more circumstantial than direct. Thus, the victim’s word is of more excellent probative value, especially for a demure woman with no apparent interest in harming the designated perpetrator of the crime”

(transcripted from the Court of Justice of São Paulo, 2019).

This case is repeated in several other cases in the São Paulo State Court of Justice. In these cases, most of the time, a woman is accusing a man of sexual assault. There is very little evidence or proof, and therefore, the judge has to rely on the word of the woman accusing. The excerpt from the judicial decision states that the word “demure woman” carries great probative weight. This excerpt exemplifies how the word of the demure woman can support or anchor the judge’s opinion. There is no need for further proof. The word of a demure woman is used as an anchor to make the point that her words must be valid and true.

### 2.4. Confirmation Bias

According to [13], confirmation bias occurs when an individual makes a specific decision based on beliefs or what they want to believe. The authors describe this as one of the most problematic existing biases.

Faced with its two systems, Ref. [12] states that System 1 is gullible and prone to believe, but System 2 oversees doubting. However, System 2 is lazy. Thus, the author demonstrates that people are more likely to be influenced when tired. In this case, associative memory contributes to confirmation bias, which will shorten decision-making when an internal search for confirming evidence occurs. Therefore, the author states that the confirmatory bias of System 1 favors the acceptance of suggestions and the exaggeration of the probability of unlikely events.

In the judicial sphere, Ref. [12] states that the rational judge will nevertheless strive for compatibility, even though internal consistency is more easily achieved and assessed. Thus, at first glance, tired and hungry judges seem more inclined to issue condemnatory judgments rather than thoroughly evaluate the evidence presented in the case. This result is found in [16] and has been disputed [17], who show that the order of the kind of cases is relevant, not lunch.

Nonetheless, there is consolidated jurisprudence of the Court of Justice of the State of São Paulo through which the words “deprecated woman, with no apparent interest in causing harm” have great decision-making weight.

### 2.5. Heuristic of Affect, Coherence, and Result Bias

As mentioned earlier, the affect heuristic predicts that people will let their likes and dislikes determine their beliefs and judgments. For example, Ref. [12] states that the individual’s attitude towards complex issues, such as pesticides in food, red meat, nuclear energy, tattoos, or motorcycles, will be superimposed on their benefits or risks. However, the author warns that, even with such pre-judgments formed, the individual’s mind is not permanently closed, and their beliefs can be changed through information and sensible argumentation. Thus, concerning attitudes, System 2 acts as a champion for System 1’s emotions. In short, active System 1 seeks coherence and suggests reasonable solutions to the complacent System 2.

When discussing coherence, Ref. [12] highlights that causality does not indicate correlation. He argues that, through System 1, individuals are prepared from birth to have impressions of causality independent of reasoning about causation patterns. However, the illusion of veracity is perceived in the individual’s ease of believing a well-told, cohesive, and coherent story. Thus, even if untrue, judges can base their decisions on a particular level.

According to [11], rational judges will look for compatibility, although internal consistency may be more easily achieved and assessed. This means that, according to the authors, they will establish a correlation between their judgments of probability and their knowledge of the subject, and they will consider the laws of probability and their heuristic and judgment biases.

Finally, we present the outcome bias through which individuals recognize the success or failure of a particular measure only after the fact. These attitudes seem prudent only when seen in retrospect. Also, according to [12], the retrospective bias will be more significant as the consequences worsen.

As the author states [12], we are prone to blame decision-makers for sound decisions that went wrong and to give them little credit for measures that seem apparent only after the fact. Imagine a hypothesis analyzed by a judge in which there is a request for compensation for the collapse of a property. The petitioner demonstrates that he had previously informed the construction company of the existence of cracks. In hindsight, it would be evident that something would happen. However, how many properties have cracks that did not lead to their collapse? Thus, they will probably have incurred this bias whenever the judge evaluates certain conduct as evident and aggravates the offender’s penalty.

## 3. Results

Given the specific case presented by the interested party, the judge must, without doubt, render a decision following Brazilian legislation, having weighed the evidence produced during the process. However, Brazilian procedural doctrine recognizes that guaranteeing the parties the right to make the relevant evidence is insufficient.

As stated by [18], after the conclusion of the procedural instruction, the judge will form their conviction according to the principle of rational persuasion. When deciding, they will be responsible for substantiating the sentence and demonstrating how they were convinced about the facts under consideration by the parties. The parties, in turn, may require the judge to pronounce precisely on the evidence produced.

Indeed, the judicial decision must be reasoned, as determined by Article 93, item IX, of the Brazilian Federal Constitution. Article 131 of the Civil Procedure Code of 1973, when dealing with the assessment of evidence, prescribed that the judge would freely assess the evidence, considering the facts and circumstances contained in the case file, even if not alleged by the parties, indicating in the decision the reasons that formed their conviction.

The current Civil Procedure Code [19], article 371, provides that the judge will examine the evidence contained in the case file, regardless of the subject who has promoted it, and will indicate in the decision the reasons for the formation of their conviction.

The expression “freely” was removed, and it was no longer possible to speak of “freely motivated conviction of the judge”. Ref. [20] states that the change guarantees the political responsibility of the judge, who is no longer the protagonist of the process. The decision can no longer be based on feeling, prejudice, or the judge’s idea. Its foundation should come from the law and not from personal perceptions. Therefore, although the judge is inevitably subject to behavioral and heuristic biases, notably those mentioned in the previous item, the need to justify the decision acts contrary to the maintenance of their personal biases.

Ref. [21] corroborates this position, which states that, even if the doctrine recognized that the “free assessment” did not allow for the assessment of evidence as the judge wanted, the suppression of the term “freely” indicates that there must be a rational assessment of the evidence.

Therefore, the suppression of the idea of “free motivated conviction” of the judge gives rise to the “justify-decide” being linked to a precise and dense script with explanatory hypotheses. Ref. [22] coined the expression “free (pseudo) motivated conviction” to indicate the harmfulness of decisions that do not show adequate reasoning.

Ref. [23] states that only based on a “relationality/instrumentality conception of the process”, even if “democratic norms” are disregarded, it would be possible to affirm a certain jurisdictional discretion, although perhaps disguised as an affirmation of the possibility of a particular free motivated conviction.

As for the example, Brazilian legislation still tries to prevent the prevalence of such biases through the prescription contained in article 489, § 1o of the Code of Civil Procedure [19].

Art. 489. The following are essential elements of the sentence:

§ 1° It is not considered reasoned any judicial decision, whether interlocutory, sentence, or judgment, which

I limit itself to the indication, reproduction, or paraphrase of a normative act without explaining its relationship with the cause or the decided issue;
II use indeterminate legal concepts without explaining the concrete reason for its incidence in the case;
III invoke reasons that would serve to justify any other decision;
IV not face all the arguments deduced in the process capable of, in theory, invalidating the conclusion adopted by the judge;
V limit itself to invoking precedent or summary statement without identifying its determining grounds or demonstrating that the case under trial fits those grounds;
VI failure to follow the statement of jurisprudence or precedent invoked by the party without demonstrating the existence of a distinction in the case under trial or the overcoming of the understanding.

Therefore, the legal provision mentions several cases in which the decision will not be reasoned, giving rise to its nullity. Ref. [18] based on other authors, emphasizes that, in addition to the impartiality sought, the need for sufficient reasoning translates constitutional guarantee, the duty of jurisdiction, rights of the parties, and even “popular participation in the administration of justice” (in the sense that the foundations of the sentence allow for control not only by the parties but also by lawyers and jurisdictions in general).

The judge will determine that the necessary evidence be produced, not simply the convenient or opportune ones (article 370 of the Code of Civil Procedure). Ref. [18] states that Brazilian procedural law hardly grants the judge freedom to decide based on convenience or opportunity. This is because judicial protection is based on the element of necessity (or utility).

As an example, we quote an excerpt from the Court of Justice of the State of São Paulo in which the judge rejects the evidentiary assessment based on the victim’s reputation (Excerpt taken from the judgment handed down in Criminal Appeal No. 0015092-37.2017.8.26.0482, October/2021):

Still, regarding the defendant’s version, it is noteworthy that he sought to attack the victim’s honor in court, pointing out that she was a prostitute. It is an argumentative resource that strives to resonate in practice correctly banned from the legal system by removing the expression “honest woman” from the Penal Code in 1940. The victim’s reputation matters little for the characterization of the crime.

On this discretion, Ref. [24] clarify that this occurs so that the judge can use his instructive powers to conduct the process actively. The authors then defend the active position of the judge conducting the procedural instruction to finalize the evidence in the best possible way.

However, although the legislation requires the decision’s reasoning, it does not adequately constrain the judge to motivate the evidentiary reason. Ref. [21] states that the normative text must be interpreted in such a way that it is concluded that the demonstration by the judge of their evidentiary reasoning that led them to the decision is an integral part of the reasoning on the facts.

Suppose he is not bound by criteria and values fixed in the law. In that case, the judge cannot disregard the rules of logic, positive sciences, and basic economic principles, as their discretion does not have an absolute character. It is clear, therefore, the attempt of the legislation for the judge to act according to the Theory of Rational Decision, previously refuted by Simon. After all, can the judge act strictly to the limit of the legislation, disregarding their biases?

As demonstrated, considering that even experienced researchers cannot wholly disregard their behavioral biases (which leads us to believe that they are present in all human beings in an irrational way), although the legislation aims to extirpate the personal influence of the decision-making process, the mere prescription of conducts and limits does not seem to be sufficient to eliminate or reduce the biases of judges, who may be utterly oblivious to their occurrence.

## 4. Discussion

As can be observed, Brazilian legislation, in the search for impartiality in the judicial sphere, prescribes several rules to honor the objective production of evidence, such as article 371 of the Code of Civil Procedure. However, even with prescriptions like this, Ref. [21] emphasizes the need to develop objective doctrinal and jurisprudential criteria as well as their evidentiary standards that define the degree of corroboration necessary for a given hypothesis to be considered proven. According to the author, a hypothesis could only be regarded as demonstrated when the evidence “can predict some empirically contrastable event or state of affairs”.

In US doctrine, there are three most common patterns of evidence: (i) preponderance of evidence; (ii) clear and convincing evidence; and (iii) proof beyond any reasonable doubt. Ref. [21] highlights the lack of impediment to creating different standards, as many as necessary. What the author finds unacceptable is that each judge considers the evidence presented to them according to their convictions.

Ref. [25] brought a list to examine decision biases. Its first part indicates the observance of the critical approach; the second indicates pre-judgments and impulsive decisions; the third indicates information processing; and the fourth analyzes the decision. We will focus on the second part, which explores behavioral biases by verifying pre-judgments and hasty decisions.

Based on the author’s suggestions, the following questions were formulated to violate pre-judgments:

(i) Will the decision-maker gain an advantage from some possible decisions? If yes, which one?
(ii) Has the decision-maker committed themselves to a particular outcome?
(iii) Is there reason to suspect that the decision-maker is biased towards any possible outcomes or that the decision-maker may influence them?
(iv) Have opposing opinions been sufficiently analyzed?

Also, based on the author’s suggestions, the following questions may verify the prematurity of decisions:

(i) Is it possible to perceive an accidental bias in the considerations discussed at the beginning?
(ii) Were opposing and alternative opinions considered?
(iii) Is there any possibility that any dissonance has not been ignored?

Not all questions will apply to the judicial area, especially when the trial takes place by a single person. However, by adapting the questions, we can assess whether or not behavioral biases and pre-judgments exist in judicial decision-making. As an example, we suggest the following adaptations:

(i) Do any of the judges, whether in the first instance or courts, have more to gain from one conclusion than another?
(ii) Was someone already committed to a conclusion (e.g., thesis defended in another court case)? Is there any reason to suspect bias?
(iii) Have all the judges sufficiently analyzed the specific case and its peculiarities?
(iv) Have decision possibilities (e.g., subsidiary requests) been fully considered? Was it the evidence in the record that led to an inevitable conclusion?
(v) Did data, uncomfortable opinions, or characteristics of the parties constitute an important influence on the decision?

Specifically, regarding the judicial area, Ref. [26] points out two strategies, such as those that have been advocated in the literature, to combat or reduce biases in judicial decision-making: (i) use of techniques to raise awareness among judges aiming at “debiasing”; and (ii) the replacement of judges by algorithms.

Ref. [27] indicates possible ways to reduce the influence of biases in the decision-making process by increasing the time available for judges’ decisions and suggesting that they start writing down their opinions about the cases during their processing to stimulate their logical and deductive judgment. The author also adds the importance of targeted training and the inclusion of checklists for elaborating a systematic process to achieve deliberative reasoning. Finally, the author argues for affirmative actions that increase diversity in decision-making spaces.

### 4.1. Use Techniques to Raise the Awareness of Judges Aiming at “Debiasing”

As for decision-makers’ awareness, Refs. [11,12] point out that heuristics can be highly economical and also usually effective. These are valuable shortcuts. However, they can lead to systematic and predictable errors. Thus, “a better understanding of heuristics and biases could improve judgments and decisions in situations of uncertainty”.

Even when considering marital relationships, Ref. [12], based on several studies, suggests that spouses who are aware of their own biases can contribute to peace in their marriage. This occurs because spouses remember their efforts and contributions much more than others.

Ref. [28], when analyzing the decisions in rape cases, also highlight the need that a first step in combating biases would be the science of these by the judges themselves as well as the knowledge of the so-called “gender bias”. In this perspective, also considering the objective production of evidence, the authors highlight some parameters that should be observed in those specific cases, such as adopting legal frameworks for women’s human rights. This is to have minimum equality for the judges, preventing them from starting their analysis from their own perspectives. Likewise, Ref. [29] argues that knowing them is the first step in combating cognitive deviations in judicial decisions.

In this context, the existence of a complete and efficient database of judicial decisions is a fundamental tool for understanding and combating biases in judicial decisions. In the United States, the JUSTFAIR system is the first large-scale, free, public criminal database that conveys information about the characteristics of defendants, their sentences, and the identity of the sentencing judge.

It was noted that JUSTFAIR sentencing records using data from the United States Sentencing Commission, the Federal Judicial Center, the Public Access Court Electronic Records system, and Wikipedia revealed that judges assign longer sentences to black, male, or low-income individuals compared to members of other racial, gender, and income groups; additional data reveal that the political affiliation of judges in federal criminal cases has an impact on the term of convictions [30].

### 4.2. The Replacement of Judges by Algorithms

According to [12], the individual must assume that any number on the table can have an anchoring effect. Therefore, considering the object’s value, the author states that the individual “must mobilize (mobilize their System 2) to combat the effect”. By bringing the lesson to the judicial area, considering that behavioral biases are intrinsic to human beings, one of the possible solutions allowed for by current technology is the use of decisions made by algorithms.

When investigating the route to automated decisions from the beginning, Ref. [31] affirm that there is a wealth of judicial decisions that illustrate instances of non-cognitivist behaviors by judges, where decisions are influenced by personal opinions that are purely subjective.

According to the authors, there is evidence of a “right of explanation and transparency” linked to algorithms, especially decision-making. However, there are still several doubts about how to implement them. After all, such algorithms must be sensitive and valuable and cannot be “black box” style. Thus, its use should occur in the most transparent way possible, enabling the explanation of decision-making. Another control mechanism that can assist in using algorithms is the one that attests to its procedural regularity, regardless of whether the result is fair (use of software). They will need to be periodically audited.

In this sense, for example, Ref. [32] built a model for predicting decisions of the Supreme Court of the United States based on a time evolution random forest classifier that leverages the engineering of unique features to predict more than 240,000 justice votes and 28,000 case outcomes over almost two centuries (1816–2015). According to the authors, a 70.2% accuracy was achieved at the case outcome level and 71.9% at the justice vote level, revealing an increase in the general level of forecasting and advancement in the science of quantitative legal forecasting.

Finally, Ref. [31] argue that using algorithms is an inescapable reality. Technological advances push us further along this path every day. Therefore, they should be implemented but with caution.

### 4.3. Review of Judicial Decisions, Including through Collegiate Bodies

Although biases are intrinsic to human beings, reviewing judicial decisions, including through collegiate bodies (various interpretations and views), can help combat personal biases. However, Brazilian law only provides for the review of the decision, called double degree of jurisdiction, when the decision rendered is contrary to the public entity (Article 496 of the Code of Civil Procedure). As for the other cases, the decision will only be reviewed if one of the parties appeals.

As for collegiate bodies, the so-called strategy of the tenth man (whose authorship is unknown), which idea is quite simple, calls attention. According to it, when a group of people must analyze something and decide, it should never be unanimous. It will be up to at least one group member to disagree with everyone else, even if this goes against their convictions. Thus, when the group looks at the hypotheses and arguments brought by the dissenter, they will have a broader view of the possibilities and, in theory, will be able to make a better decision (even if the conclusion is a confirmation of the initial decision).

Similarly, Ref. [13] cite the philosophical rule that the hypotheses presented must be tested by scientists seeking to refute them. Only in this way will it be possible to ascertain its degree of probability.

Ref. [28] state that making the composition of the Judiciary more heterogeneous would also support the review of decisions with the ideal of combating personal biases since other points of view would be analyzed. The implementation of affirmative measures and the process of diversification in the Judiciary are also defended, which would help to reduce “bias implicit in the Judiciary” due to the greater diversity of thoughts.

## 5. Conclusions

The present article investigates at what stage the Brazilian literature relates to behavioral and heuristic biases in judicial decision-making. Through a discussion on judicial decision-making, we notice that the following are commonly identified in judicial decisions: the heuristic of representativeness, the heuristic of availability, the heuristic of anchoring (anchoring effect), the confirmation bias, and the heuristic of affection, coherence, and the resulting bias. These biases, although intrinsic to human beings, influence the due impartiality of the judge.

Brazilian legislation aims to reduce the judge’s influence and prescribes the judge’s conduct, especially the need to justify their conclusion. However, as observed, the rules are not enough to eliminate behavioral biases intrinsic to human beings.

Finally, by agreeing that the reduction in behavioral biases in judicial decision-making is essential, the literature presents some solutions that could be adopted for their mitigation, among them are (i) the use of techniques to raise awareness of judges aiming at debiasing; (ii) the replacement of judges by algorithms (computerization); and (iii) the review of court decisions, including through collegiate bodies.

We present some evidence that cognitive biases may be present in judicial decisions. We contribute to the theoretical studies on the subject by highlighting the biases and heuristics commonly identified in judicial decisions and the attempts of Brazilian legislation to mitigate them. We also contribute to a practical analysis by highlighting the initiatives already adopted to combat them.

Algorithms stand out among the options that can be used. This is because, as [31] mention, using automated decisions, considering technological advances, is inevitable. According to them, given the more excellent verification of positive than negative results, it will be necessary to face the risks so that the scientific discussion, guided by the postulates of ethics, leads to the use of technological resources in the judicial sphere. However, it is essential to highlight that algorithms have to be trained and can also be a source of biases since they are trained on existing real decisions by “imperfect” human decision-makers. These potential biases have to be taken into account before using algorithms to improve judicial decision-making.

The present study analyzed only the Brazilian literature on the subject. Given its objective of specifying the most common biases and heuristics in judicial decisions, it was limited to analyzing judicial decisions. Therefore, future research with a larger territorial scope is suggested, which may influence the species of biases and heuristics. Developing nudges to help debias judicial decisions would also be an important step (see [33]).

Furthermore, in addition to judicial decision-making, the approach by [34] stands out. Through this approach, the knowledge of behavioral biases in judgment—especially the priming effect, the halo effect, anchoring, negligence with the base rate, and the framing effects—may also improve mediation and conciliation.

We acknowledge some limitations of this paper. Our focus is on how cognitive biases may worsen judicial decisions, and as such, we can observe the mitigation or elimination of these cognitive biases as the main goal of a judicial system. Ref. [35] takes a different approach and shows that cognitive biases may work as they have helped humans make decisions over time in a Darwinian sense. Further research could exploit this approach and evaluate judicial decisions under this new insight. A second aspect worth mentioning is that cognitive biases may affect different laypeople and professionals (judges, lawyers, and other professionals). When groups make decisions, we should also expect the mitigation of these biases [36].

## Data Availability

Data are contained within the article.
